# Vitreoretinal Lymphoma: a clinical case highlighting the diagnostic and therapeutic challenge and the need for a multidisciplinary management

**DOI:** 10.1007/s00277-026-07122-w

**Published:** 2026-06-12

**Authors:** María Sanchidrián Mayo, Almudena Cabero Martínez, Luis Cacharro Moras, David Alonso Castronuño, María García Álvarez, Óscar Javier Blanco Muñez, Alejandro Martín García Sancho, Nora Carmen Gutiérrez Gutiérrez

**Affiliations:** 1 Ophtalmology Department, University Hospital of Salamanca, Salamanca, Spain; 2 Hematology Department, Institute of Biomedical Research of Salamanca (IBSAL), University Hospital of Salamanca, Salamanca, Spain

**Keywords:** Intraocular lymphoma, Keratouveitis, Vitritis, Methotrexate, Rituximab

## Abstract

Vitreoretinal Lymphoma (VRL) is a rare ocular malignancy often presenting with atypical retinal findings, which can lead to misdiagnosis. We report the case of a patient with bilateral intraocular lesions and reduced visual acuity. Diagnosis was confirmed by multimodal imaging and molecular analysis of aqueous humor. Treatment consisted of intravitreal methotrexate and rituximab, followed by systemic chemotherapy, achieving partial resolution of the ocular lesions but subsequent ocular and systemic recurrence. After rescue treatment, patient achieved sustained complete remission. Early diagnosis and a multidisciplinary approach are essential, and multimodal imaging combined with molecular diagnostics is key in confirming the diagnosis.

## Case presentation

A 78-year-old woman presented with progressive bilateral visual loss over several months. Her medical history was significant for rheumatoid arthritis treated with corticosteroids and methotrexate.

Initial ophthalmologic evaluation showed a visual acuity of 0.1 in the right eye and 0.05 in the left eye, normal intraocular pressure, and mild diffuse retinal pigment epithelium alterations with peripapillary atrophy.

**Image. 1:**
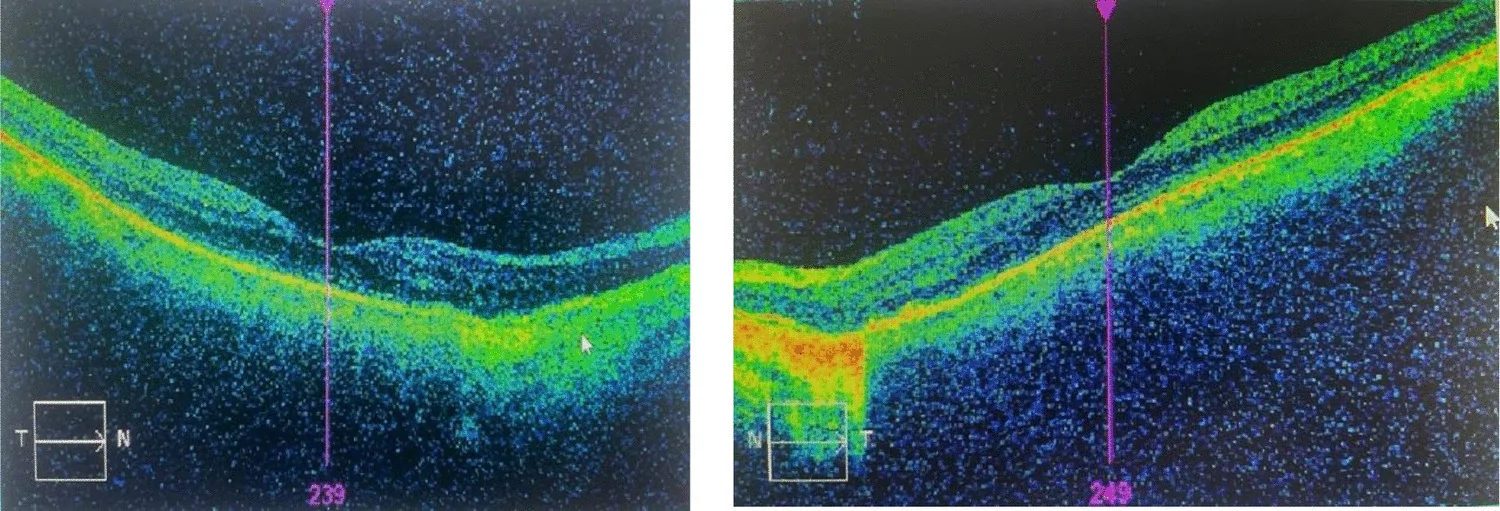
Optical coherence tomography (OCT) of right and left eye. Perimacular disruption of the retinal pigment epithelium with increased signal hypertransmission

Electroretinography revealed central retinal involvement in both eyes, while visual evoked potentials remained normal. Optical coherence tomography (OCT) demonstrated a perimacular disruption of the retinal pigment epithelium with increased signal hypertransmission (Image 1).

After four months of follow-up, the patient developed a palpable extraocular mass affecting the superior rectus muscle of the left eye, causing hypotropia and impaired supraversion. Surgical excision revealed diffuse large B-cell lymphoma (DLBCL), stage IA according to PET-CT (brain MRI did not show disease), with an R-IPI score of 1. She received four cycles of R-miniCHOP (every 3 weeks) achieving complete a metabolic response on post-treatment evaluation after two months.

**Image. 2:**
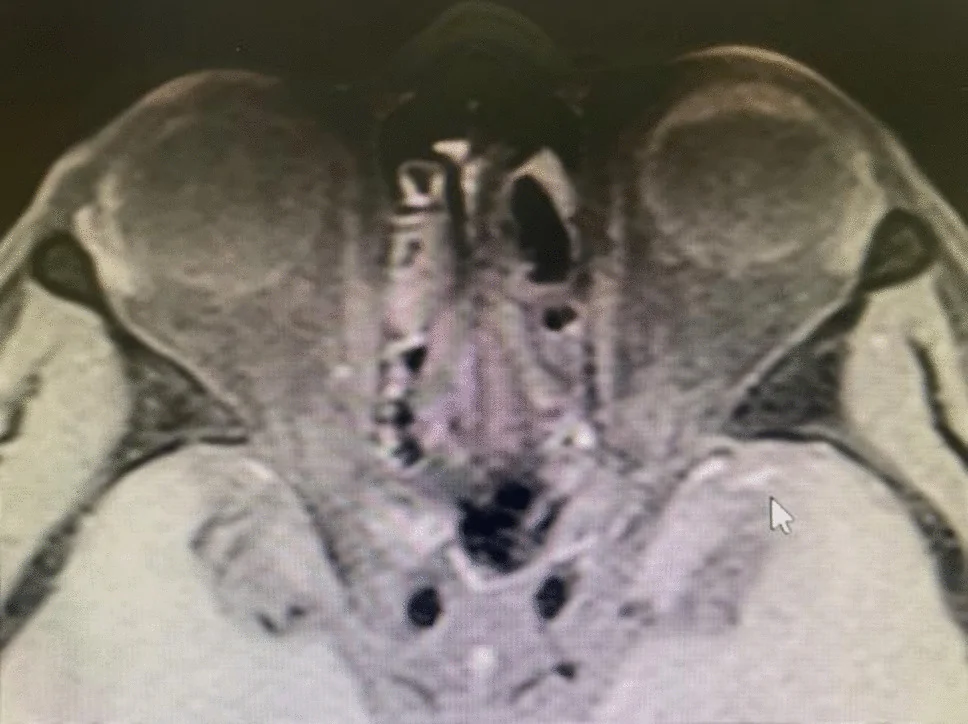
Orbital magnetic resonance: infiltration of the anterior ocular wall in the left eye. PET-CT: marked metabolic progression of the bone lesions

Six months after treatment completion, the patient presented with left eye keratouveitis. A presumed herpetic etiology prompted treatment with topical and systemic corticosteroids and antivirals, with initial resolution. One month later, she developed recurrent keratouveitis accompanied by iris surface undulations, vitritis, and serous choroidal and retinal detachment. MRI revealed infiltration of the anterior ocular wall of the left eye (Image 2). After anterior chamber paracentesis, molecular studies confirmed B-cell clonality, although *MYD88* mutation analysis was inconclusive due to insufficient sample material. These findings supported a diagnosis of probable vitreoretinal B-cell lymphoma. A vitreous biopsy was recommended but refused by the patient.

Given her age, absence of systemic or CNS involvement, and negative cerebrospinal fluid testing, she was treated with local intravitreal methotrexate (0,4 mg/ml × 3 doses) and rituximab (1 mg/0,1 ml × 2 doses) in two months according to the protocol. During therapy, she developed pupillary block and iris neovascularization, with intraocular pressure rising to 50 mmHg. Despite topical hypotensive treatment, neovascular glaucoma progressed, ultimately resulting in amaurosis. Enucleation was performed, and pathology confirmed DLBCL with optic nerve invasion.

**Image. 3:**
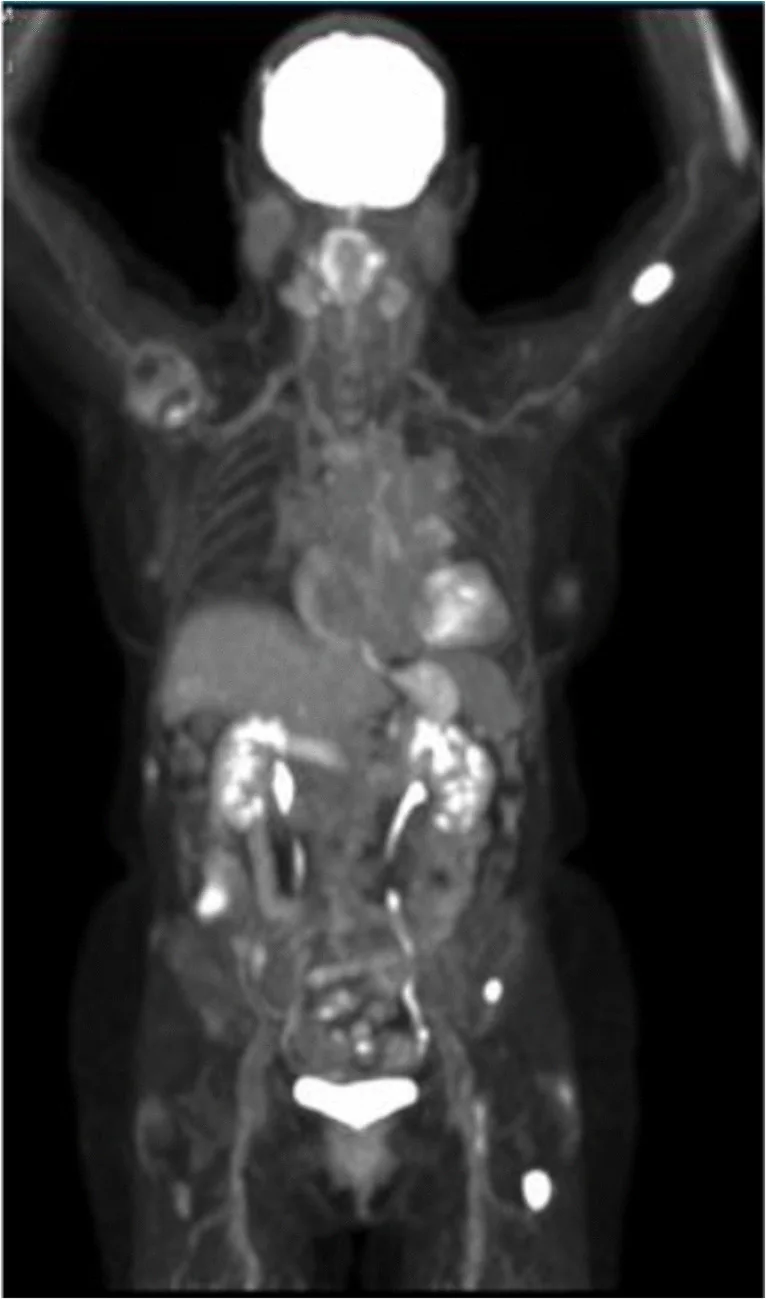
Orbital magnetic resonance: infiltration of the anterior ocular wall in the left eye. PET-CT: marked metabolic progression of the bone lesions

Subsequent one month later brain MRI suggested leptomeningeal involvement, although cerebrospinal fluid analysis remained negative. PET-CT demonstrated metabolic progression in the left periorbital region. Owing to progression limited to the optic nerve region, systemic therapy with high-dose methotrexate (1.5 g/m^2^) combined with ibrutinib was started. After two cycles, PET-CT showed a complete metabolic response in the orbit but new, nonspecific bone hypermetabolic foci (humeral diaphysis and cranial vault). A follow-up PET-CT one month later revealed orbital recurrence and clear progression of the bone foci (Image 3). A biopsy of one of these lesions confirmed relapsed DLBCL, and ibrutinib was discontinued.

Second-line immunochemotherapy with R-GemOx (rituximab, gemcitabine and oxaliplatin) was administered for four cycle (every 2 weeks), achieving complete metabolic response on PET-CT and MRI, with cerebrospinal fluid studies remaining persistently negative. The patient remains in remission. Given the elevated risk of systemic or CNS relapse, lenalidomide maintenance was initiated, but was discontinued early due to intolerance.

## Discussion

Vitreoretinal lymphoma (VRL) is a subtype of CNS lymphoma, with an incidence under 0.05 cases per 100,000 individuals. Most cases are of B-cell origin and may involve the vitreous, retina, retinal pigment epithelium, or optic nerve. VRL may occur as a primary intraocular disease or in association with systemic lymphoma [1]. Up to 25% of patients with CNS lymphoma develop intraocular involvement, and more than half of individuals with primary VRL progress to CNS disease within 30 months, highlighting the need for coordinated management between ophthalmology and hematology [2].

The disease often presents with nonspecific symptoms, such as gradual visual decline or subtle retinal abnormalities, which may mimic inflammatory or degenerative conditions. In this case, probably bilateral involvement manifested as central retinal dysfunction and perimacular disruption of the retinal pigment epithelium with increased signal hypertransmission on OCT, potentially reflecting incipient infiltrative disease. The subsequent development of an orbital mass and later severe intraocular relapse illustrates the heterogeneous and unpredictable progression of this malignancy.

The behavior of the lymphoma in this patient directly bears on the still-unresolved question of the origin of DLBCL confined to the central nervous system and to the intraocular compartment as an extension of the CNS. In this case, the first diagnosis of DLBCL was established on biopsy of an extraocular mass, and subsequently B-cell clonality was detected by PCR in the aqueous humor, confirming that the ophthalmologic findings were due to lymphomatous infiltration. Therefore, this case raises the question of where the lymphoma originated. From the standpoint of clinical evolution, one might think that the lymphoma originated in the intraocular compartment and later spread to the orbital region and, afterward, to other osseous sites. However, given how uncommon systemic relapses of primary vitreoretinal lymphoma are, it seems more consistent to suggest that systemic disease was already present before the onset of orbital symptoms and went undetected because imaging studies had not been performed.

Optic nerve infiltration, as observed in this patient, is particularly relevant, since the optic nerve represents an extension of the CNS and may serve as a route for tumor spread. Diagnosis is frequently challenging, and delays of several months or longer are typical. Imaging techniques such as MRI and PET-CT have limited sensitivity for early intraocular infiltration; therefore, imaging findings must be interpreted in conjunction with clinical suspicion and confirmed by identification of B-cell markers in the eye [3].

Definitive diagnosis relies on cytologic, immunophenotypic studies, and molecular analysis of vitreous or aqueous samples [4]. Vitreoretinal biopsy is considered the gold standard, although small sample volumes and patchy infiltration often limit diagnostic yield. Aqueous humor analysis can be helpful in selected cases. Cytology alone has modest sensitivity, while flow cytometry and PCR-based clonality assessment significantly improve diagnostic accuracy. Elevated IL-10 and IL-10/IL-6 ratio are potential biomarkers for intraocular lymphoma [5], although these markers could not be evaluated in this case.

Treatment strategies depend on age, functional status, comorbidities, and CNS involvement. High-dose methotrexate-based systemic therapy remains the cornerstone for CNS or combined CNS-ocular disease, often alongside rituximab [6]. Local therapy -typically intravitreal methotrexate or rituximab- is an appropriate option for elderly or frail patients, or in cases limited solely to the eye [7]. However, intravitreal chemotherapy carries risks, including keratopathy, intraocular inflammation, and, less commonly, neovascular glaucoma, which may result in irreversible vision loss and enucleation, as occurred in this patient.

Combined systemic and local therapy may prolong time to recurrence, but does not clearly improve overall survival. Novel agents, including Bruton tyrosine kinase inhibitors and immunomodulatory drugs such as lenalidomide, have shown activity in relapsed or refractory disease [8, 9], although responses are variable. In this patient, combined methotrexate and ibrutinib proved partially ineffective.

Monitoring treatment response at the ocular level relies on repeated ophthalmologic examinations, though these may be subjective and observer dependent. Multimodal imaging-including OCT, autofluorescence, ocular ultrasound, and fundus photography-offers non-invasive and reproducible tools [10] to aid diagnosis and follow-up. Fluorescein angiography and neuroimaging complement these modalities when clinically indicated. Surveillance of the contralateral eye is crucial due to frequent bilateral involvement, and periodic CNS imaging and lumbar puncture are recommended to detect secondary spread.

This case illustrates the diagnostic complexity and variable clinical trajectory of vitreoretinal lymphoma, emphasizing the importance of timely suspicion, multidisciplinary collaboration, and individualized therapeutic planning. Reporting such cases contributes to a deeper understanding of this uncommon malignancy and may help refine future diagnostic and therapeutic approaches.

## Data Availability

No datasets were generated or analysed during the current study.
